# Elastic Properties and Piezoelectric Energy Harvesting of a Lead-Free Hybrid Perovskite, (DABCO)RbBr_3_

**DOI:** 10.3390/molecules31061013

**Published:** 2026-03-18

**Authors:** Yiming Liu, Guangyue Shi, Changliang Li, Feng Luo

**Affiliations:** Tianjin Key Laboratory for Rare Earth Materials and Applications, Center for Rare Earth and Inorganic Functional Materials, School of Materials Science and Engineering, Nankai University, Tianjin 300350, China; 1120220591@mail.nankai.edu.cn (Y.L.); sgy@mail.nankai.edu.cn (G.S.); 1120220583@mail.nankai.edu.cn (C.L.)

**Keywords:** lead-free hybrid perovskite, elastic properties, piezoelectric energy harvesting

## Abstract

Mechanical properties of lead-free hybrid perovskites have attracted growing interest because of their significance in future eco-friendly optoelectronic applications. However, there are very limited studies about the intrinsic elastic properties and high-pressure structural evolution of hybrid perovskites, and the fundamental structure–mechanical property relationships are insufficiently understood. Here, we report the elastic behavior of a three-dimensional (3D) hybrid organic–inorganic perovskite, (DABCO)RbBr_3_ (DABCO = triethylenediammonium), and confirm the processability through processing with chiral metasurfaces and the generation of circular dichroism. Our in situ high-pressure synchrotron X-ray diffraction experiments demonstrate that this crystal does not start to amorphize until 2.3 GPa. Density functional theory calculations reveal that its *E*, *G* and *v* range between 20.73 and 27.93 GPa, 8.21 and 11.62 GPa and 0.18–0.39, respectively. Additionally, due to the low elastic moduli and polar crystal structure, we fabricate a device of (DABCO)RbBr_3_ composite film, which shows favorable performance for piezoelectric energy harvesting. This work utilizes (DABCO)RbBr_3_ to open up new avenues for applications in manufacturing and energy harvesting.

## 1. Introduction

The unprecedented rise of hybrid organic–inorganic perovskites (HOIPs) has fundamentally reshaped the landscape of solution-processable semiconductors, achieving power conversion efficiencies that rival those of traditional silicon-based technologies [1,2,3,4]. However, the commercial deployment of these materials is hindered by the acute toxicity of lead (Pb) and their inherent environmental instability [5]. Consequently, the pursuit of lead-free alternatives has become a central theme in materials science [6,7,8,9]. Among these, Rubidium (Rb)-based hybrid halides have emerged as a distinct class of chemically interesting materials. Unlike the 3D networks typical of Pb-based systems, Rb-based perovskites often crystallize in low-dimensional architectures. To maintain a 3D structure and ensure charge conservation, divalent organic cations and monovalent alkali metal ions such as Rb^+^ can be used [10,11,12]. This combination can also maintain a 3D structure rather than a low-dimensional sheet or chain structure, as in ((3-ammoniopyrrolidinium)RbBr_3_) [13]. In this category of perovskite structures, each alkali metal ion is coordinated by six adjacent halide ions, creating a distorted octahedron, while organic cations occupy the interstitial cavities to uphold overall charge neutrality. Recently, experimental and theoretical investigations have demonstrated that Rb-based perovskites possess promising properties for applications in piezoelectricity, second harmonic generation responses, optoelectronic devices and thermoelectrics [14,15,16,17,18,19,20].

Given their wide range of potential industrial applications, the mechanical properties of these perovskites are crucial, as they determine their reliability and durability during service [21,22,23,24,25]. In addition, the intrinsic mechanical properties and structural evolution of Rb-based hybrids under extreme conditions remain significantly underexplored. Understanding the mechanical behavior of these hybrid lattices is paramount for practical device engineering, as it determines their reliability and durability during processing and operation [26,27,28,29]. High-pressure research provides a powerful, clean methodology for probing these properties by tuning interatomic distances and modifying electronic orbitals without introducing chemical impurities [30]. While the mechanical properties of many 3D hybrid perovskites have been investigated, studies focusing on the fundamental structure–mechanical property relationships in Rb-based systems are rare. This paper systematically studies the mechanical properties of Rb-based perovskites with an ABX_3_ structure, which are extremely similar to (DABCO)RbBr_3_ (DABCO = C_6_H_14_N_2_). Here, we perform density functional theory calculations to analyze their elastic modulus and demonstrate (DABCO)RbBr_3_ processability by a high-precision focused ion beam. In situ synchrotron high-pressure X-ray diffraction (HP-XRD) experiments were performed to examine the high-pressure behavior of the materials and to monitor the evolution of their lattice parameters under compression. More importantly, we fabricate a device based on (DABCO)RbBr_3_ composite film and explore its piezoelectric energy harvesting. These findings provide critical insights into the rigidity and stability of Rb-based hybrids, paving the way for the design of mechanically robust, lead-free functional materials.

## 2. Results and Discussion

**Figure 1:**
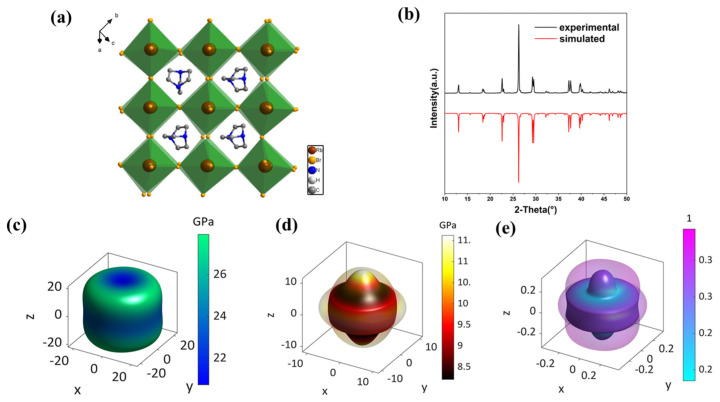
(**a**) 3D packing structure of (DABCO)RbBr_3_. (**b**) PXRD pattern of (DABCO)RbBr_3_, with black representing experimental and red representing simulated. 3D representations of (**c**) Young’s moduli, (**d**) shear moduli and (**e**) Poisson’s ratios of (DABCO)RbBr_3_.

(DABCO)RbBr_3_ crystallizes in the hexagonal crystal system with the non-centrosymmetric space group *P*322_1_. As shown in Table S1, the cell parameters are a = b = 9.6444 Å, c = 23.254 Å and unit cell volume V = 1873.2 Å^3^. As shown in Figure 1a and Figure S1a, the inorganic framework consists of RbBr_6_ octahedra, with each Rb^+^ coordinated to six adjacent Br^−^. These octahedrons further extend into a perovskite framework, while the perovskite cavities are occupied by protonated DABCO^2+^ cations, thus forming the [RbBr_3_]^2−^ framework. There are abundant hydrogen bonds between the organic amine and the inorganic halide framework, as shown in Figure S1b. The N-H…Br hydrogen bond distances range from 2.244 Å to 2.28 Å, with bond angles between 169.9 and 170.6°. The experimental powder X-ray diffraction pattern (PXRD) matches well with the calculated XRD pattern, demonstrating the phase purity of our sample (Figure 1b). First-principles calculations were conducted to determine its electronic band structure and density of states (DOS). As shown in Figure S2, it possesses an indirect bandgap structure at the Γ point in the Brillouin zone with a bandgap value of 4.55 eV. The valence band maximum and conduction band minimum (CBM) are primarily dominated by Br-4p Rb-4d orbitals, respectively (Figure S2b).

The elastic properties of crystals are fundamental to elucidating their piezoelectric behavior and to assessing their fatigue performance under practical operating conditions. We calculated the full elastic stiffness tensor (C*_ij_*) matrix using DFT (Table S2). It is worth noting that C_14_ has an exceptionally small value. Although (DABCO)RbBr_3_ exhibits trigonal symmetry due to the orientation of organic cations, its macroscopic mechanical framework fully behaves like a highly symmetric hexagonal crystal system. The *P*322_1_ space group structure lacks the “sixfold rotation axis” of a hexagonal crystal system; this lower symmetry introduces a sixth independent elastic constant *C*_14_. Based on the C*_ij_* matrix, we derived the Young’s modulus (*E*), shear modulus (*G*), Poisson’s ratio (*v*) and sound velocities, as presented in both 3D and 2D representations. The maximum value (*E*_max_ = 27.93 GPa) is observed along the [2-12] direction, while the minimum value (*E*_min_ = 20.73 GPa) occurs along the [001] direction (*c*-axis) in Figure S3. The anisotropy index *A_E_* = *E*_max_/*E*_min_ = 1.35 shows that the anisotropy is moderate. The observation that the *c*-axis is the softest direction suggests that the bonding interactions or packing density along the *c*-axis are weaker. This is probably because there are fewer coordination bonds and weaker hydrogen–bond interactions. The anisotropy in mechanical behavior facilitates a deeper understanding of the anisotropic photoconductivity driven by the vertical crystallite orientation in quasi-2D perovskites [31]. Similarly, the *G*, which represents resistance against shear deformation, ranges from 8.21 GPa to 11.62 GPa, with an anisotropy index of 1.42 in Figure 1c. The minimum shear modulus along the [01-1] direction is clearly described in Figure S4. The Poisson’s ratios, which describe transverse expansion relative to axial compression, are considered. As shown in Figure 1d and Figure S5, the calculated Poisson’s ratio values for (DABCO)RbBr_3_ range from 0.18 along the [01-1] direction to 0.39 along the [0-11] direction. In this context, much larger stress was needed for generating the same deformation along [01-1] in comparison to that along [0-11]. Consequently, the strain along [0-11] was exceedingly larger than that along [01-1], which led to a maximum ν along [0-11] and a minimum one along [01-1].

**Figure 2:**
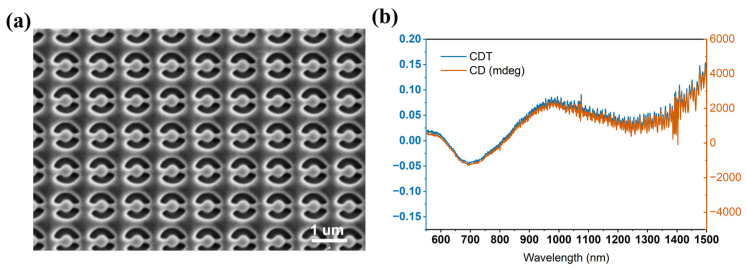
Scanning electron microscopy image (**a**) and Experimental CD (**b**) of patterned (DABCO)RbBr_3_ film.

Based on the above-noted elastic properties, the relatively moderate stiffness of (DABCO)RbBr_3_ indicates that the lattice can accommodate local strain more readily than hard ceramic and perovskite oxides. Therefore, we further explored its processability in nano fabrication. As shown in Figure 2a, periodic planar chiral open ring metasurfaces are directly formed on a (DABCO)RbBr_3_ thin film obtained by thermal evaporation using a high-precision focused ion beam (FIB). The Scanning electron microscopy image shows a pattern without cracks, and the chiral units are well separated and the overall periodicity is consistent. We also performed the FIB process in a (DABCO)RbBr_3_ single crystal, as shown in Figure S6; the single crystal remained intact as well. These results indicate that the elastic properties of (DABCO)RbBr_3_ are favorable for the FIB process. The transmittance spectra of the metasurfaces under LCP and RCP light incidence are shown in Figure S7; the calculated circular dichroism (CD) values are shown in Figure 2b. The CD can therefore be defined as CD = (*T*_RCP_ − *T*_LCP_)/(*T*_RCP_ + *T*_LCP_) and expressed in millimeters; CD, *θ* (mdeg), can also be defined as

(1) $$\theta \left(mdeg\right)=\frac{180,000}{\pi }\arctan\left(\frac{\sqrt{T_{RCP}}-\sqrt{T_{LCP}}}{\sqrt{T_{RCP}}+\sqrt{T_{LCP}}}\right)$$

Figure 2b shows chirality over a wide range (600–1500 nm), reaching 3000 mdeg in milliseconds at 1000 nm, indicating the successful fabrication of our chiral metasurfaces.

The FIB process is accompanied by ion bombardment and possible local disorder near the milled edges. To further evaluate the intrinsic structural tolerance of (DABCO)RbBr_3_ under compression and to understand its tendency toward disordering, we carried out in situ high-pressure synchrotron X-ray diffraction (HP-XRD) measurements. Upon compression, all diffraction peaks shift continuously toward higher angles, indicating whole lattice shrinkage and no apparent phase transition (Figure S9a,c). As shown in Figure S9b, the c-axis contracts by 5.0%, while the a-axis contracts by 4.2%. These findings suggest that the c-axis is more readily compressible than the a- and b-axes, which can be attributed mainly to the relatively small Rb–Br–Rb bond angle and the comparatively weak hydrogen-bonding interactions along this direction. In addition, the unit cell volume for (DABCO)RbBr_3_ shows a reduction of 10.7% (Figure S9d). The unit cell volume–pressure data obtained prior to the onset of amorphization were fitted using a second-order Birch–Murnaghan equation of state. The obtained bulk modulus (*B*) of (DABCO)RbBr_3_ is 21.6 GPa. This experimental value matches exceptionally well with the theoretical bulk modulus of 22.4 GPa, validating the accuracy of our computational model. There is a relatively low bulk modulus compared to fully inorganic perovskites like Cs_2_AgBiBr_6_ [32]. Organic DABCO components are softer than all-inorganic ones, thus enhancing the material’s low mechanical strength.

**Figure 3:**
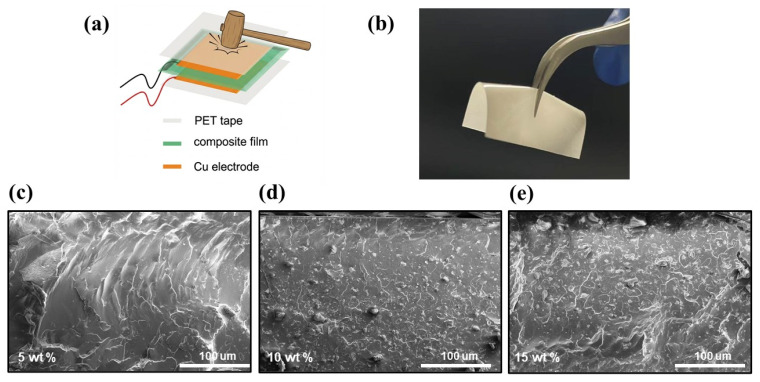
(**a**) Schematic representation of a Piezoelectric energy harvesting device based on Cu/composite film/Cu. Optical image of 10 wt% (DABCO)RbBr_3_/PDMS composite film (**b**) and different concentrations in a cross-sectional SEM image (**c**–**e**).

The relatively low elastic moduli and polar crystal structure indicate that (DABCO)RbBr_3_ has great potential for energy conversion applications and can realize mechanical–electric conversion processes through a piezoelectric response. To further explain the piezoelectric behavior of (DABCO)RbBr_3_, a series of (DABCO)RbBr_3_/PDMS composite films were prepared with a weight ratio (wt%) ranging from 5% to 10% to 15%. The prepared films were then sandwiched between two copper plates to form a sandwich structure and encapsulated with polyethylene terephthalate (PET) tape to fabricate the device, as shown in Figure 3a and Figure S10. The optical photograph of the 10 wt% composite film in Figure 3b clearly shows that the film with an area of 2 × 2 cm^2^ can be easily folded and twisted. Therefore, under a given pressure, a larger strain can be induced in a flexible film, which is also beneficial for the harvesting of piezoelectric energy. We quenched the composite film and imaged its cross-section using a scanning electron microscope (SEM). Figure 3c–e show the cross-sections of the composite film at 5, 10 and 15 wt%, respectively. It is clear that as the concentration increases, (DABCO)RbBr_3_ gradually accumulates within the composite film.

**Figure 4:**
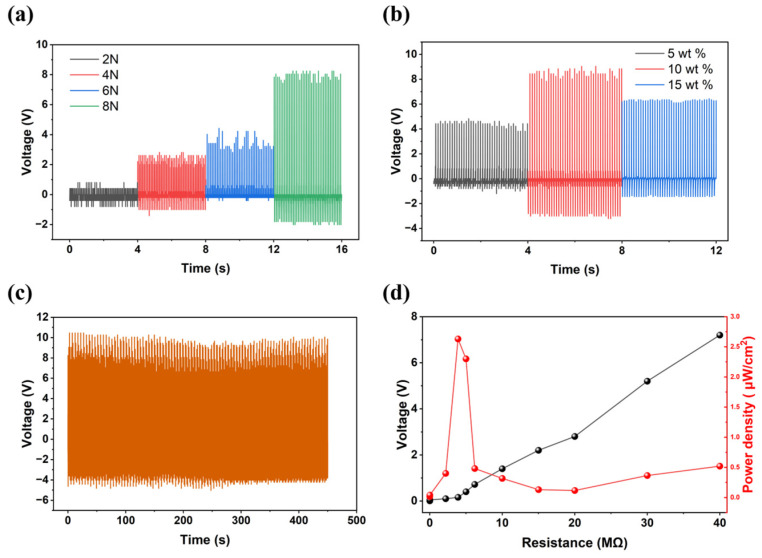
Piezoelectric properties of (DABCO)RbBr_3_/PDMS devices. (**a**) Voltage of 5 wt% composite film with increasing pressure. (**b**) Voltage of composite films with different concentrations. (**c**) Voltage of composite film for 30 days under laboratory conditions within 450 s cycles. (**d**) Voltage and power density as functions of load resistance.

**Figure 5:**
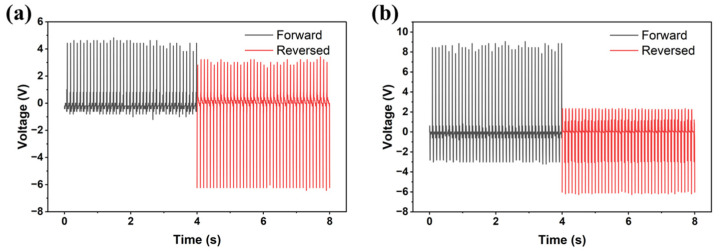
Piezoelectric properties of (DABCO)RbBr_3_/PDMS devices. Voltage of 5 wt% (**a**) and 10 wt%. (**b**) Composite films when flipping the electrodes.

The piezoelectric energy harvesting process was recorded using an oscilloscope under periodic impact. We selected a 5 wt% composite film and tested the piezoelectric output performance by varying the applied stress. When the stress gradually increased from 2 N to 8 N at a frequency of 10 Hz, the signal value gradually increased from 0.4 V to 8 V (Figure 4a), demonstrating that the device exhibited a sensitive response even under minimal stress. We further tested the output performance at three different concentrations, as shown in Figure 4b. The voltage increased as the concentration increased from 5 wt% to 10 wt% but decreased again as the concentration continued to increase to 15 wt%. Based on the SEM observations in Figure 3e, we observed excessive aggregation of the piezoelectric microparticles. Such aggregation leads to an inhomogeneous stress distribution and poor interfacial stress transfer from the polymer matrix to the piezoelectric particles. Furthermore, the randomly oriented dipoles in the agglomerated clusters can cause a localized cancellation of macroscopic polarization, resulting in a reduction in overall piezoelectric output [33,34]. The device underwent a 450s cycle test after being placed in laboratory conditions for 30 days, demonstrating that the piezoelectric device has good environmental stability (Figure 4c). This robust stability is attributed to the effective isolation from atmospheric moisture provided by the PDMS and PET layers. The voltage of the 5 wt% composite film was measured under external load resistances ranging from 1 kΩ to 40 MΩ, and the power density was calculated to reach a maximum of 2.6 μW/cm^2^ at a load resistance of 3.9 kΩ (Figure 4d). The piezoelectric output voltage and power density of the (DABCO)RbBr_3_ device and other reported lead-free hybrid piezoelectric devices are summarized in Table S3. When compared with peers under the same low-force (~2 N), (DABCO)RbBr_3_ remains competitive. To verify that these signals originate from the piezoelectric effect rather than the triboelectric effect, we selected devices with concentrations of 5 wt% and 10 wt% for electrode reversal test, as shown in Figure 5a,b. The device’s voltages also exhibited opposite signals with similar amplitudes after electrode reversal.

## 3. Experimental Section

Materials and Synthesis: (DABCO)RbBr_3_ were synthesized through simple solvent evaporation according to the reported methods [11]. In total, 1 mmol of triethylenediamine (DABCO) and 1 mmol of RbBr (both purchased from Macklin, Shanghai, China) were dissolved in a mixed solvent system consisting of 10 mL hydrobromic acid and 10 mL ethanol in a 50 mL beaker. After several days, high-quality colorless block crystals precipitated at the bottom of the vessel (Figure S11).

Crystal data collection and refinement: Powder X-ray diffraction (PXRD) (Rigaku, Tokyo, Japan) measurements were performed to verify phase purity using a Rigaku MiniFlex 600 diffractometer. The scan range was set from 3 degrees to 50 degrees with a step size of 0.02 degrees and a scan speed of 5 degrees/min. The experimental diffraction pattern showed excellent agreement with the simulated pattern derived from single-crystal data, indicating high phase purity without detectable impurities.

Single-crystal diffractions of (DABCO)RbBr_3_ were performed on a Rigaku XtaLAB MM007 CCD diffractometer (Cu Kα λ = 1.5418 Å) (Rigaku, Tokyo, Japan). The final structures were solved using Olex2 software, V1.5 [35,36] based on the ShelXT-2016 [37] with an intrinsic phasing method and refined through the ShelXL program. All non-hydrogen atoms were refined anisotropically, and the hydrogen atoms were located and refined geometrically.

Computational Methodology: First-principles calculations were conducted using the CASTEP code within the framework of Density Functional Theory (DFT) [38]. The crystal geometry was fully optimized using the Broyden–Fletcher–Goldfarb–Shanno (BFGS) algorithm prior to property calculation [39]. The elastic stiffness constants (C*_ij_*) were determined using the finite strain method [40]. A plane-wave basis set with a cutoff energy of 450 eV was employed. All calculations were performed in reciprocal space under non-spin-polarized conditions to accurately derive the band structure, density of states (DOS) and elastic moduli.

Metasurface fabrication and circular dichroism measurement: The arrays of metasurfaces were patterned on the film and single crystal, with a Helios 5 CX Focused Ion Beam system (Thermo Scientific, Waltham, MA, USA), using a nominal beam current of 30 kV, 40 pA.

The measurements were performed on a custom-designed optical characterization system. White light from a supercontinuum light source (NKT photonics, Birkerød, Denmark) was converted into fixed-wavelength light with high intensity and high collimation by a spectrometer. After passing through an objective lens (PAL-10-NIR), the beam was focused onto the sample surface to form a very small spot, and the transmission spectrum of the structure was measured. The data then needed to be normalized with respect to air.

Synchrotron high-pressure X-ray diffractions (HP-XRD): In situ HP-XRD experiments of the two compounds were performed on the 4W2 beamline at the Beijing Synchrotron Radiation Facility (BSRF, Beijing, China). The wavelength of the X-ray beam was 0.6199 Å. The diamond anvil cell (DAC) with a culet diameter of 400 μm was applied to exert hydrostatic pressure. Fully ground powder samples were loaded into an approximately 120 μm diameter hole, which is pre-indented in the stainless-steel gasket with a thickness of about 40 μm. Silicone oil was used as the pressure-transmitting medium and ruby spheres were employed for pressure calibration. The diffraction patterns were recorded on a Pilatus 2M detector and integrated using the FIT2D software package, V10.3 [41]. Le-Bail fitting was conducted using the Total Pattern Solution (TOPAS) software v4.2.0 [42] to obtain the cell parameters under different pressures.

Preparation of (DABCO)RbBr_3_/PDMS flexible composite films: In total, 2.5 g of PDMS precursor was mixed with different amounts of (DABCO)RbBr_3_ micro powders (5, 10 and 15 wt%), and then 15 mL of dichloromethane (CH_2_Cl_2_) was added. The mixture was stirred for 4 h to remove the dichloromethane. Afterward, a curing agent was added to the liquid and stirred for 0.5 h to ensure curing. Subsequently, the mixture was placed in a vacuum-drying oven to remove air bubbles generated during stirring. Finally, the film precursor prepared using a doctor blade was placed in an oven at 100 °C for 1 h to form a flexible film.

Fabrication of the piezoelectric energy harvesting device: A thin film was cut into 2 × 2 cm^2^ dimensions to serve as the piezoelectric source, and two copper foils were attached to the film as the top and bottom electrodes. Subsequently, two copper wires were adhered to the electrodes to detect the output signal. Finally, the device was encapsulated with PET tape to prevent the intrusion of water or other solvents.

## 4. Conclusions

In summary, we conducted a study on the structure, electronic and mechanical properties of lead-free organic–inorganic hybrid perovskite (DABCO)RbBr_3_ as a model. The material has a three-dimensional structure and a broad indirect bandgap of 4.55 eV, which is caused by the [RbBr_3_]^2−^ inorganic framework. Our combined theoretical and experimental approach revealed significant mechanical anisotropy, pinpointing the crystallographic c-axis as the axis of highest compressibility. Chiral metasurfaces were fabricated using FIB and circular dichroism was successfully generated in the near-infrared wavelengths. The mechanical behavior was further verified through HP-XRD experiments. Furthermore, we fabricated a device based on (DABCO)RbBr_3_ composite film which shows good piezoelectric energy harvesting properties. These findings not only demonstrate the potential of Rb-based hybrids as ductile alternatives to toxic Pb-based materials but also provide fundamental physical insights into their behavior under extreme mechanical environments and their potential for energy harvesting applications.

## Data Availability

The data supporting the findings of this study are available from the corresponding authors upon reasonable request.

## Supplementary Materials

The following supporting information can be downloaded at: https://www.mdpi.com/article/10.3390/molecules31061013/s1, Figure S1: 3D packing structure and hydrogen bonding of (DABCO)RbBr_3_ (a and b); Figure S2: Band gap and density of states of (DABCO)RbBr_3_ (a and b); Figure S3: 2D representations of Young’s moduli; Figure S4: 2D representations of shear moduli; Figure S5: 2D representations of Poisson’s ratios; Figure S6: Scanning electron microscopy image in a single crystal of (DABCO)RbBr_3_; Figure S7: Transmittance under left-handed and right-handed circularly polarized light of (DABCO)RbBr_3_; Figure S8: Refined X-ray diffraction pattern of (DABCO)RbBr_3_; Figure S9: In-situ high-pressure XRD pattern (a) and diffraction rings (b) during compression at a selected pressure of (DABCO)RbBr_3_. (DABCO)RbBr_3_ cell parameters as a function of pressure: Axial compressibility (c) and changes in unit cell volume with pressure (d); Figure S10: Piezoelectric energy harvesting device based on Cu/(DABCO)RbBr_3_ composite film/Cu; Figure S11: optical photograph of (DABCO)RbBr_3_; Table S1: Comparison of cell parameters of (DABCO)RbBr_3_; Table S2: The elastic constant matrix C_ij_ of (DABCO)RbBr_3_; Table S3: The piezoelectric output performance of common lead-free hybrid piezoelectric composite film materials [43,44,45,46,47,48].
