# Supplementary material for: Elastic Properties and Piezoelectric Energy Harvesting of a Lead-Free Hybrid Perovskite, (DABCO)RbBr3
Source: Molecules. 2026 Mar 18;31(6):1013. doi: 10.3390/molecules31061013 (PMC13029141; doi:10.3390/molecules31061013)
Supplement: Supplementary file 1 [file molecules-31-01013-s001.zip › molecules-4169757-supplementary.pdf]

Electronic Supplementary Information

Elastic Properties and Piezoelectric Energy Harvesting of a Lead-Free Hybrid Perovskite, (DABCO)$RbBr_3$

Yiming Liu, Guangyue Shi, Changliang Li, Feng Luo*

School of Materials Science and Engineering, Nankai University, Tianjin 300350, China.
* Correspondence: feng.luo@nankai.edu.cn

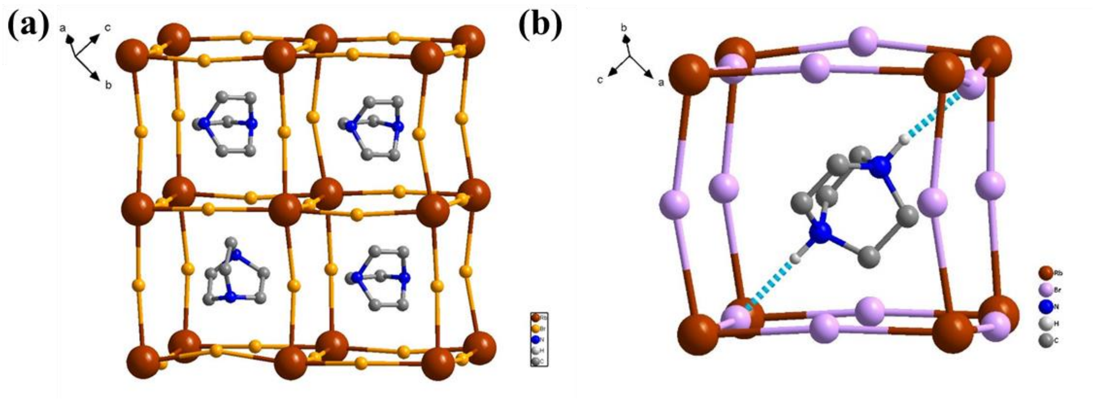

Figure S1. 3D packing structure and hydrogen bonding of (DABCO)$RbBr_3$ (a and b).

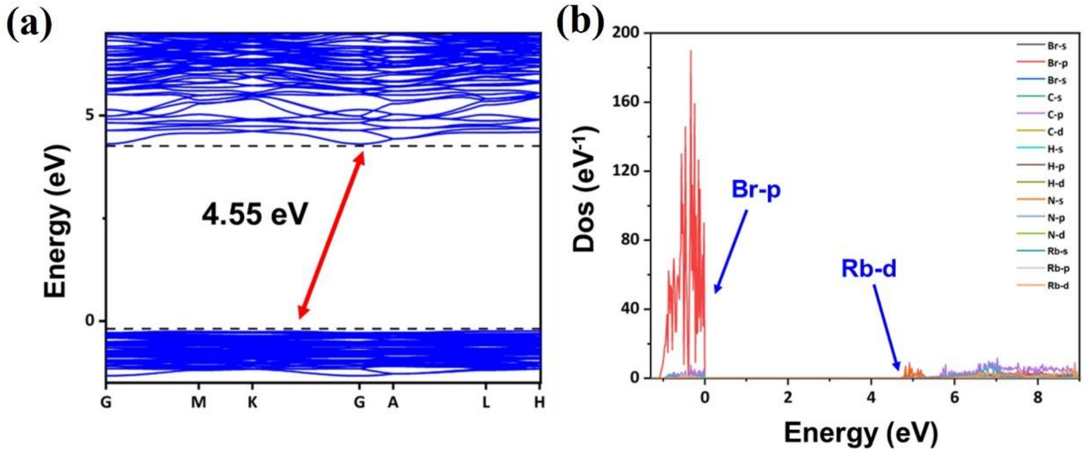

Figure S2. Band gap and density of states of (DABCO)$RbBr_3$ (a and b).

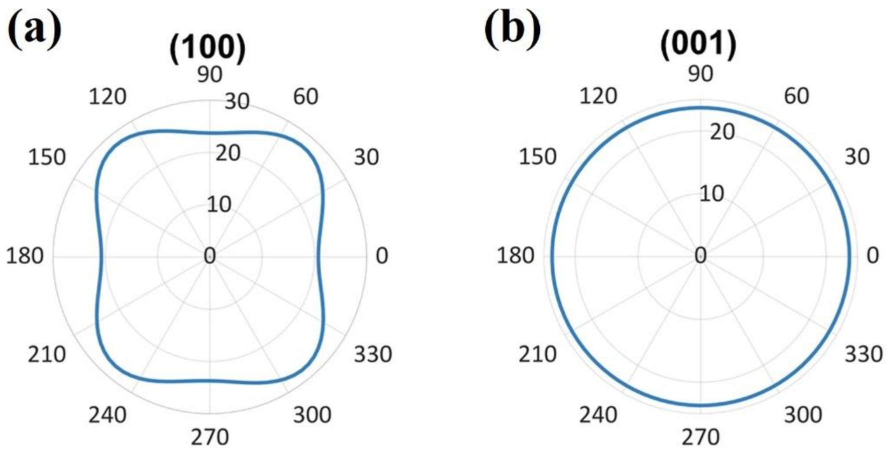

Figure S3. 2D representations of Young's moduli.

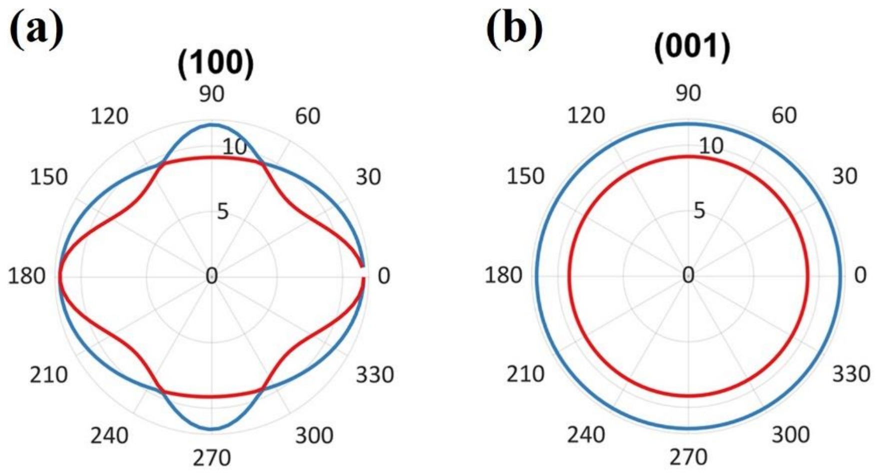

Figure S4. 2D representations of shear moduli.

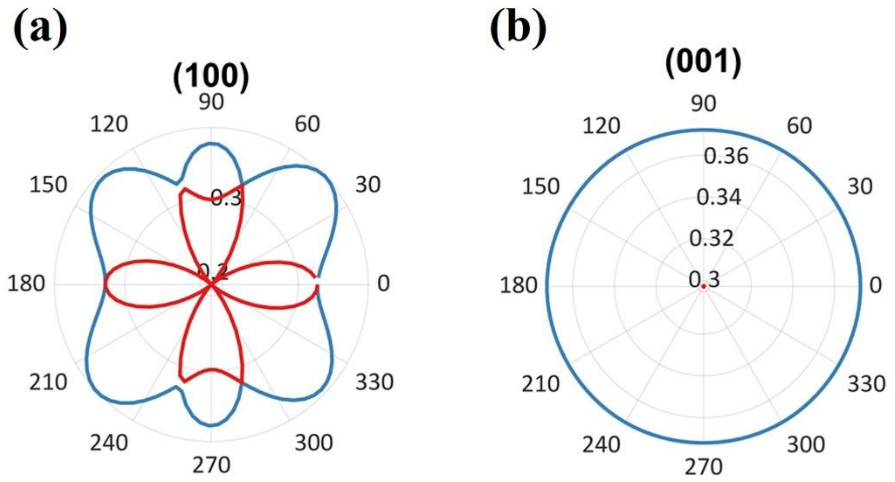

Figure S5. 2D representations of Poisson’s ratios.

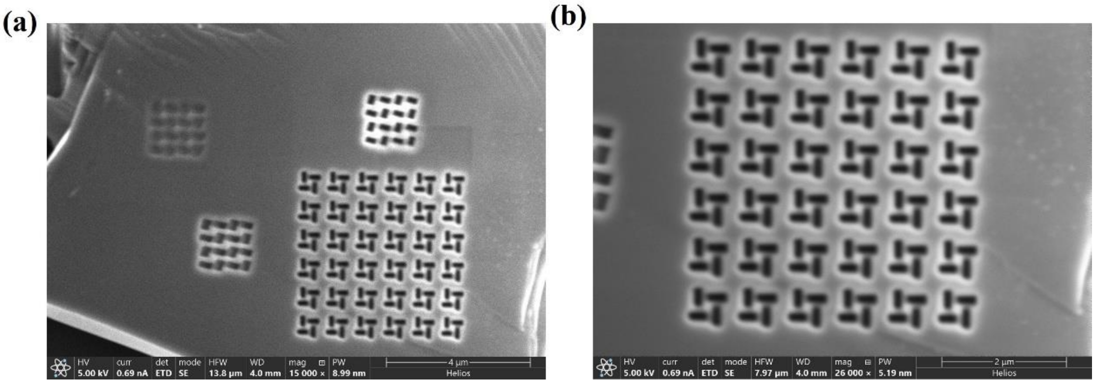

Figure S6. Scanning electron microscopy image in a single crystal of $(DABCO)RbBr_3$.

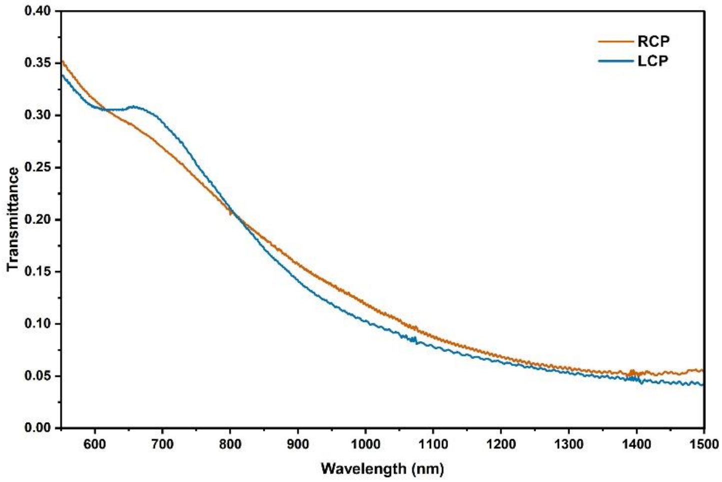

Figure S7. Transmittance under left-handed and right-handed circularly polarized light of (DABCO)$RbBr_3$.

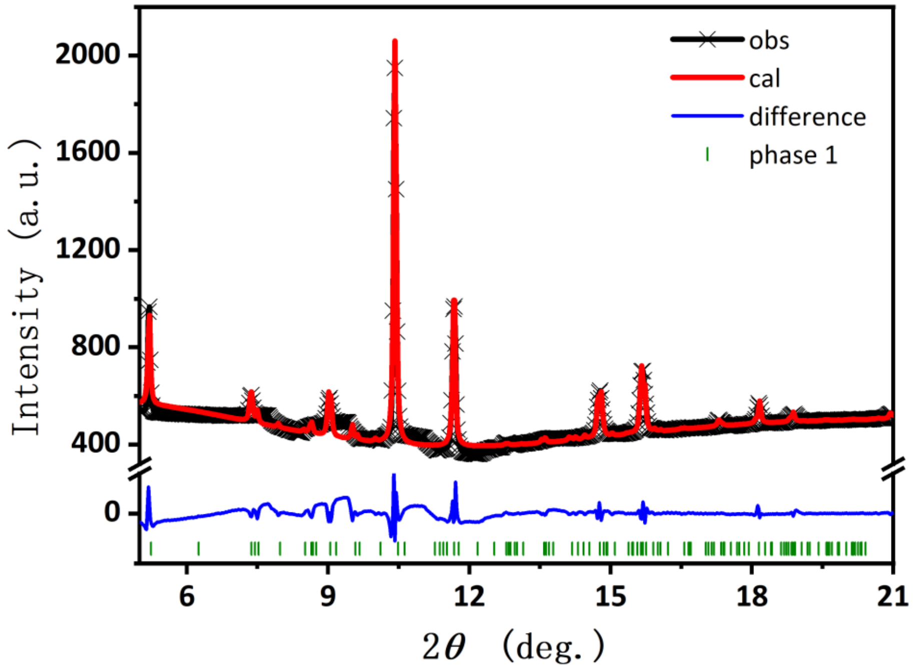

Figure S8. Refined X-ray diffraction pattern of (DABCO)$RbBr_3$

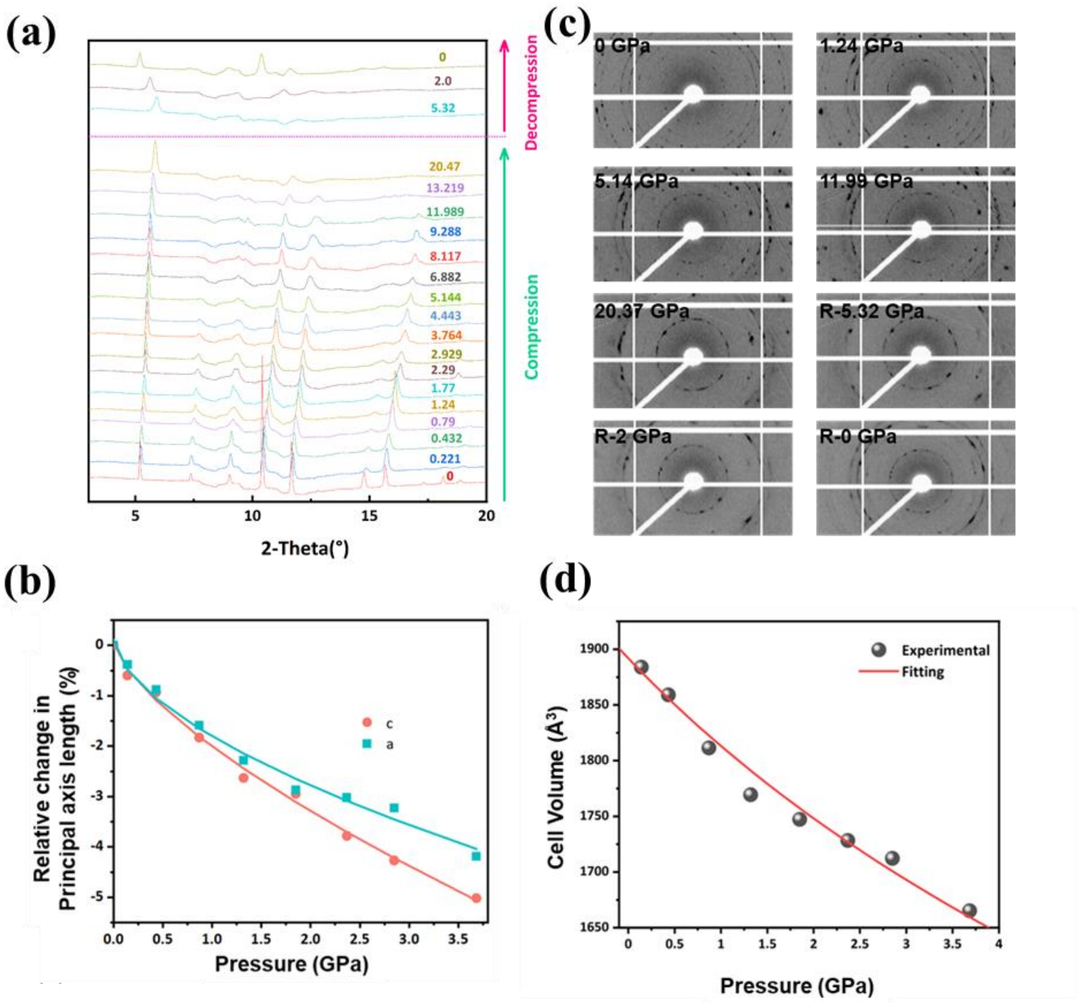

Figure S9. In-situ high-pressure XRD pattern (a) and diffraction rings (b) during compression at a selected pressure of (DABCO)$RbBr_3$. (DABCO)$RbBr_3$ cell parameters as a function of pressure: Axial compressibility (c) and changes in unit cell volume with pressure (d).

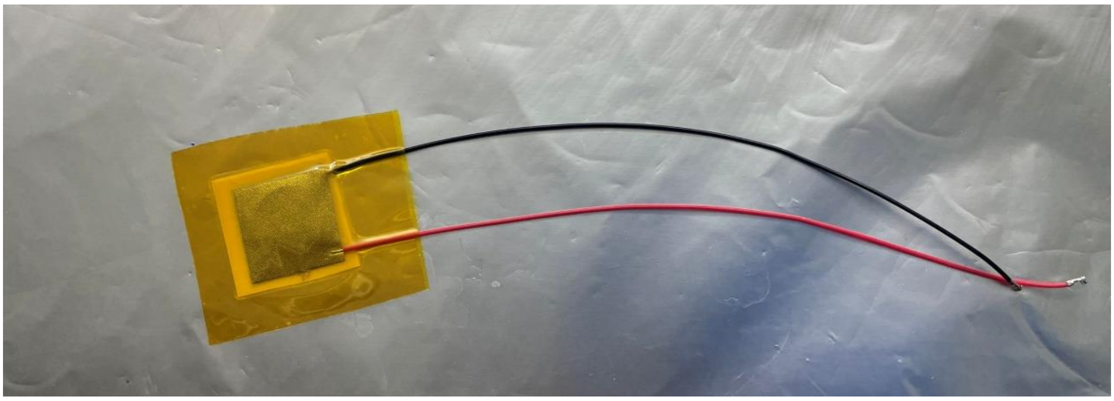

Figure S10. Piezoelectric energy harvesting device based on Cu/(DABCO)$RbBr_3$ composite film/Cu.

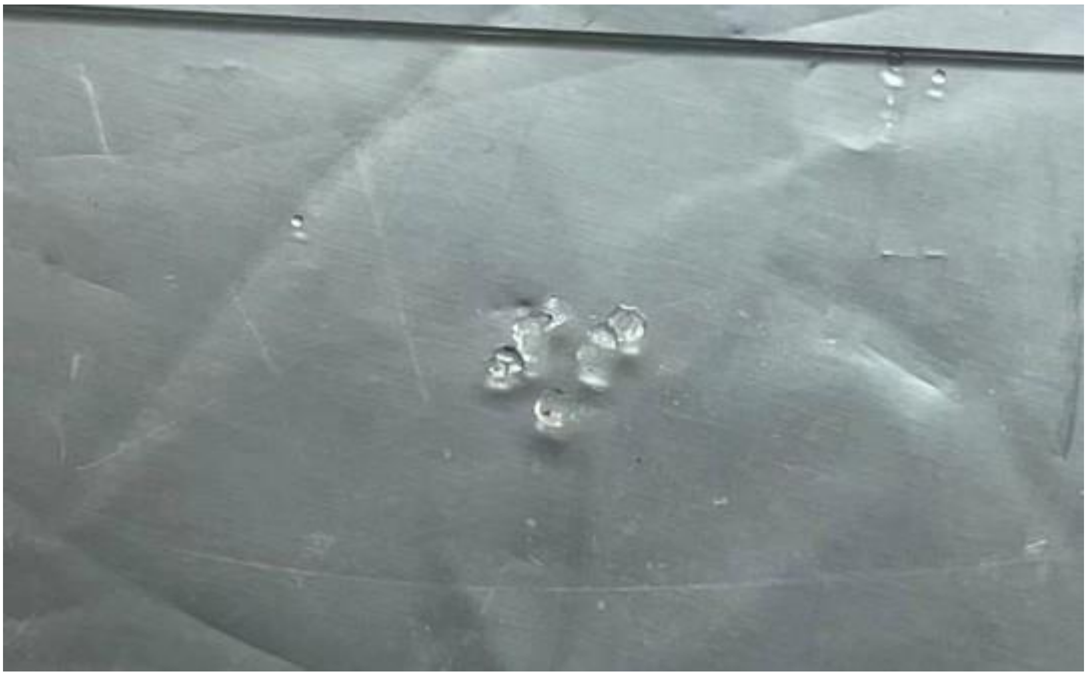

Figure S11. optical photograph of (DABCO)$RbBr_3$.

Table S1 Comparison of cell parameters of $(DABCO)RbBr_3$

	$(DABCO)RbBr_3$
Formula	$(C_6H_{14}N_2)RbBr_3$
Mr (g mol^{-1})	439.36
Space group	$P322_1$
Crystal system	hexagonal
a (Å)	9.6444 (6)
b (Å)	9.6444 (6)
c (Å)	23.254(12)
V (Å^3)	1873.2(7)
Z	6
F(000)	1236.0
Dc (gcm^{-3})	2.337
μ (mm^{-1})	13.522
R_{int}	0.0652
Limiting indices	$-11 \le h \le 12$<br>$-19 \le k \le 19$
GOF on F^2	1.025
R_1, wR_2 [$I>2\sigma(I)$][a]	0.0203 0.0470

[a] $R = \Sigma||F_o| - |F_c|| / \Sigma|F_o|$; [b] $R_w = [\Sigma[w(F_o^2 - F_c^2)^2] / \Sigma w(F_o^2)^2]^{1/2}$.

Table S2 The elastic constant matrix C_{ij} of $(DABCO)RbBr_3$

C_{ij}（GPa）	$(DABCO)RbBr_3$
C_{11}	34.87125
C_{33}	31.646
C_{44}	11.61975
C_{12}	16.608
C_{13}	16.7602
C_{14}	0.0792

Table S3 The piezoelectric output performance of common lead-free hybrid piezoelectric composite film materials.

Materials	Force (N)	Voltage (V)	Power density ($\mu W/cm^2$)	Refs.
$(DABCO)RbBr_3$	2	8.5	2.6	this work
$R\text{-}(MP)CdCl_4$	2	2.57	0.55	43
$S\text{-}(APP)_2CoBr_4$	2	10.21	3.07	44
$(BTMA)_2CoBr_4$	2.5	19.7	11.72	45
$(BPMBDMA)(Bi_2Br_9)$	21	24.6	13.65	46
$(Me_3NCH_2CH_2OH)CdCl_3$	40	55.2	70.9	47
$(DPDP)PF_6$	15	8.5	1.74	48

Reference

43. An, L.-C.; Zhao, C.; Zhao, Y.; Zhang, Y.; Li, K.; Stroppa, A.; Li, W.; Bu, X.-H., Chiral 1D hybrid metal halides with piezoelectric energy harvesting and sensing properties. *Small Structures* **2023,** *4* (11), 2300135.

44. Yang, H.-R.; Zhao, C.; Huang, S.-S.; Zhang, Z.; Wei, X.-Q.; Wang, G.-Z.; Guo, T.-M.; Feng, R.; Li, W.; Bu, X.-H., Chiral lead-free 0D hybrid metal halides for piezoelectric energy harvesting and underwater ultrasound detection. *Chinese Chemical Letters* **2025**, 111176. DOI: 10.1016/j.cclet.2025.111176

45. Guo, T. M.; Gong, Y. J.; Li, Z. G.; Liu, Y. M.; Li, W.; Li, Z. Y.; Bu, X. H., A new hybrid lead-free metal halide piezoelectric for energy harvesting and human motion sensing. *Small* **2022,** *18* (3), 2103829.

46. Meena, N.; Sahoo, S.; Deka, N.; Gadagin, V. B.; Zaręba, J. K.; Boomishankar, R., Ferroelectricity and piezoelectric energy harvesting of an $A_3M_2X_9$-type 0D bromobismuthate hybrid with a bulky organic quaternary amine. *Dalton Transactions* **2025,** *54* (7), 2985-2990.

47. Shi, C. H.; Deng, S. Y.; Li, J. Y.; Chen, H. R.; Cao, Q. L.; Zhong, W. H.; Zhu, J. Y.; Shao, S.; Bao, W.; Du, H., Structural Phase Transition-Associated Ferroelectricity and Piezoelectric Energy Harvesting in 0D Halogenozincate Hybrid via Cation's Terminal Group Lengthening. *Small Methods* **2025,** *9* (8), 2500118.

48. Vijayakanth, T.; Srivastava, A. K.; Ram, F.; Kulkarni, P.; Shanmuganathan, K.; Praveenkumar, B.; Boomishankar, R., A flexible composite mechanical energy harvester from a ferroelectric organoamino phosphonium salt. *Angewandte Chemie* **2018,** *130* (29), 9192-9196.
